# FTO stabilizes MIS12 and counteracts senescence

**DOI:** 10.1007/s13238-022-00914-6

**Published:** 2022-04-06

**Authors:** Sheng Zhang, Zeming Wu, Yue Shi, Si Wang, Jie Ren, Zihui Yu, Daoyuan Huang, Kaowen Yan, Yifang He, Xiaoqian Liu, Qianzhao Ji, Beibei Liu, Zunpeng Liu, Jing Qu, Guang-Hui Liu, Weimin Ci, Xiaoqun Wang, Weiqi Zhang

**Affiliations:** 1 State Key Laboratory of Brain and Cognitive Science, CAS Center for Excellence in Brain Science and Intelligence Technology, Institute of Brain-Intelligence Technology (Shanghai), Institute of Biophysics, Chinese Academy of Sciences, Beijing, 100101 China; 2 State Key Laboratory of Membrane Biology, Institute of Zoology, Chinese Academy of Sciences, Beijing, 100101 China; 3 University of Chinese Academy of Sciences, Beijing, 100049 China; 4 Institute for Stem Cell and Regeneration, Chinese Academy of Sciences, Beijing, 100101 China; 5 Beijing Institute for Stem Cell and Regenerative Medicine, Beijing, 100101 China; 6 CAS Key Laboratory of Genomic and Precision Medicine, Beijing Institute of Genomics, Chinese Academy of Sciences, Beijing, 100101 China; 7 China National Center for Bioinformation, Beijing, 100101 China; 8 Advanced Innovation Center for Human Brain Protection, and National Clinical Research Center for Geriatric Disorders, Xuanwu Hospital, Capital Medical University, Beijing, 100053 China; 9 Aging Translational Medicine Center, International Center for Aging and Cancer, Xuanwu Hospital, Capital Medical University, Beijing, 100053 China; 10 Chongqing Renji Hospital, University of Chinese Academy of Sciences, Chongqing, 400062 China; 11 Sino-Danish College, University of Chinese Academy of Sciences, Beijing, 101408 China; 12 State Key Laboratory of Stem Cell and Reproductive Biology, Institute of Zoology, Chinese Academy of Sciences, Beijing, 100101 China; 13 Beijing Institute for Brain Disorders, Beijing, 100069 China

**Dear Editor,**

N^6^-methyladenosine (m^6^A) is an abundant epitranscriptomic modification that regulates messenger RNA (mRNA) biology. The m^6^A modification regulates mRNA splicing, transport, stability, and translation through coordinated activities by methyltransferases (writers), binding proteins (readers), and demethylases (erasers) (Huang et al., 2020; Wu et al., 2020). Among m^6^A regulators, fat mass of obesity-associated protein (FTO), is the first discovered eraser with RNA m^6^A demethylation activity (Jia et al., 2011). Since then, FTO has been reported to play m^6^A-dependent roles in a variety of physiological processes including adipogenesis, neurogenesis and tumorigenesis (Fischer et al., 2009; Li et al., 2017; Huang et al., 2020). Consequently, FTO deficiency in mice leads to dramatic phenotypes, such as decreased fat mass and impaired brain development (Fischer et al., 2009; Li et al., 2017). Similarly, inhibition of FTO reduces tumorigenesis in multiple types of cancer models, while FTO is highly expressed in many cancers (Huang et al., 2020).

Although FTO is primarily linked with biological functions related to demethylation of internal m^6^A, recent work indicates a more complex role in FTO-mediated epitranscriptional regulation. Specifically, FTO was reported to demethylate other substrates, such as N^6^,2′-O-dimethyladenosine (m^6^A_m_) and N^1^-methyladenosine (m^1^A), and, based on its varied cellular distribution across cell types, proposed to be afforded differential access to RNA substrates (Wei et al., 2018; Sun et al., 2021). Indeed, in recent work, we discovered that m^6^A RNA methylation is reduced in human stem cell models of senescence, and that m^6^A-dependent mRNA stabilization of MIS12, a kinetochore component, protects against senescence (Wu et al., 2020). However, whether and how FTO regulates human stem cell homeostasis remain largely unexplored. Here, using CRISPR/Cas9-based strategy, we generated FTO-deficient human stem cell models and demonstrated an m^6^A-independent way by which FTO stabilizes MIS12 and antagonizes human stem cell senescence.

To investigate the role of FTO in regulating human stem cell homeostasis, we first generated FTO-deficient human embryonic stem cells (hESCs) through CRISPR/Cas9-based gene editing (Fig. 1A and 1B). FTO depletion was verified by western blotting and immunofluorescence staining (Figs. 1C, 1D and S1A). Furthermore, we did not detect any off-target effects resulting from FTO deletion (Fig. S1B). Although a previous study reported potential large structural variations near the target site induced by CRISPR-editing in hESCs (Bi et al., 2020), karyotype and copy number variation analyses revealed that the genomic integrity of FTO-deficient (*FTO*^−/−^) hESCs was well maintained (Fig. S1C–F). Relative to wild type (WT, *FTO*^+/+^) hESCs, DNA hypomethylation at the *OCT4* promoter region and the expression of typical pluripotency markers, including OCT4, SOX2 and NANOG, were comparable in *FTO*^−/−^ hESCs (Figs. 1E, S1G and S1H). Immunofluorescence staining and flow cytometry analysis further showed comparable Ki67-positive cell numbers and cell cycle status between *FTO*^+/+^ and *FTO*^−/−^ hESCs (Fig. 1F and 1G). Similarly, we did not detect any remarkable differences in the expression of nuclear lamina-associated protein LAP2 and heterochromatin relevant proteins HP1α and H3K9me3, whose downregulation was associated with stem cell senescence (Shan et al., 2021; Li et al., 2022), between *FTO*^+/+^ and *FTO*^−/−^ hESCs (Figs. 1H and S1I–K). Altogether, these data indicate that FTO is dispensable for maintaining hESC self-renewal and the expression of pluripotency genes.

**Figure 1:**
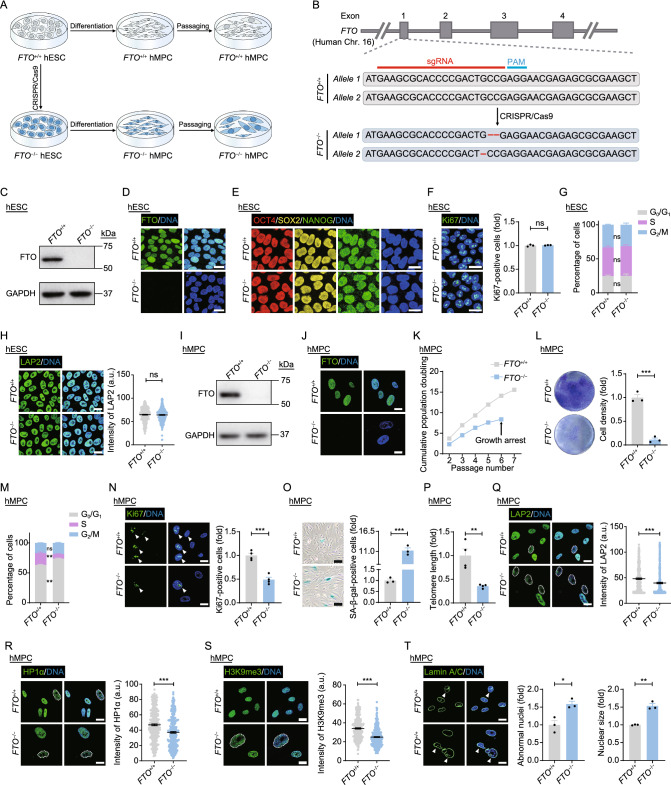
**Generation and phenotypic analyses of FTO**^**-/-**^ **hESCs and hMPCs**. (A) Schematic diagram showing the generation of *FTO*^−/−^ hESCs and hMPCs. (B) Schematic illustration of *FTO* gene editing strategy using CRISPR/Cas9-mediated non-homologous end-joining (NHEJ) in hESCs. Grey boxes indicate the exons of *FTO* gene. The red bold line indicates the sgRNA sequence. CRISPR/Cas9-mediated gene editing resulted in 1-bp (G) deletion in one allele and 2-bp (CC) deletion in another allele of *FTO* gene. (C) Western blot analysis of FTO in *FTO*^+/+^ and *FTO*^−/−^ hESCs. GAPDH was used as the loading control. (D) Immunofluorescence analysis of FTO in *FTO*^+/+^ and *FTO*^−/−^ hESCs. Scale bars, 25 μm. (E) Immunofluorescence analysis of pluripotency markers OCT4, SOX2 and NANOG in *FTO*^+/+^ and *FTO*^−/−^ hESCs. Scale bars, 20 μm. (F) Immunofluorescence analysis of Ki67 in *FTO*^+/+^ and *FTO*^−/−^ hESCs. Scale bars, 25 μm. Data are presented as the means ± SEM. *n* = 3 biological replicates. ns, not significant. (G) Cell cycle analysis of *FTO*^+/+^ and *FTO*^−/−^ hESCs. Data are presented as the means ± SEM. *n* = 3 biological replicates. ns, not significant. (H) Immunofluorescence analysis of LAP2 in *FTO*^+/+^ and *FTO*^−/−^ hESCs. Scale bars, 20 μm. Data are presented as the means ± SEM. *n* > 300 cells from three biological replicates. ns, not significant. (I) Western blot analysis of FTO in *FTO*^+/+^ and *FTO*^−/−^ hMPCs. GAPDH was used as the loading control. (J) Immunofluorescence analysis of FTO in *FTO*^+/+^ and *FTO*^−/−^ hMPCs. Scale bars, 20 μm. (K) Growth curve analysis of *FTO*^+/+^ and *FTO*^−/−^ hMPCs. Data are shown as one representative from three independent experiments with similar results. (L) Clonal expansion analysis of *FTO*^+/+^ and *FTO*^−/−^ hMPCs. Data are presented as the means ± SEM. *n* = 3 biological replicates. ***, *P* < 0.001. (M) Cell cycle analysis of *FTO*^+/+^ and *FTO*^−/−^ hMPCs. Data are presented as the means ± SEM. *n* = 3 biological replicates. ns, not significant; **, *P* < 0.01. (N) Immunofluorescence analysis of Ki67 in *FTO*^+/+^ and *FTO*^−/−^ hMPCs. Scale bars, 20 μm. White arrows indicate Ki67-positive cells. Data are presented as the means ± SEM. *n* = 4 biological replicates. ***, *P* < 0.001. (O) SA-β-gal staining of *FTO*^+/+^ and *FTO*^−/−^ hMPCs. Scale bars, 100 μm. Data are presented as the means ± SEM. *n* = 3 biological replicates. ***, *P* < 0.001. (P) Telomere length analysis of *FTO*^+/+^ and *FTO*^−/−^ hMPCs. Data are presented as the means ± SEM. *n* = 4 biological replicates. **, *P* < 0.01. (Q) Immunofluorescence analysis of LAP2 in *FTO*^+/+^ and *FTO*^−/−^ hMPCs. Scale bars, 20 μm. White dashed lines indicate the nuclei with diminished signals of LAP2. Data are presented as the means ± SEM. *n* > 300 cells from three biological replicates. ***, *P* < 0.001. (R) Immunofluorescence analysis of HP1α in *FTO*^+/+^ and *FTO*^−/−^ hMPCs. Scale bars, 20 μm. White dashed lines indicate the nuclei with diminished signals of HP1α. Data are presented as the means ± SEM. *n* > 300 cells from three biological replicates. ***, *P* < 0.001. (S) Immunofluorescence analysis of H3K9me3 in *FTO*^+/+^ and *FTO*^−/−^ hMPCs. Scale bars, 20 μm. White dashed lines indicate the nuclei with diminished signals of H3K9me3. Data are presented as the means ± SEM. *n* > 300 cells from three biological replicates. ***, *P* < 0.001. (T) Immunofluorescence analysis of Lamin A/C in *FTO*^+/+^ and *FTO*^−/−^ hMPCs. Scale bars, 20 μm. White arrows indicate abnormal nuclei. Statistical results of the relative percentage of abnormal nuclei and relative nuclear size are presented as the means ± SEM. *n* = 3 biological replicates. *, *P* < 0.05; **, *P* < 0.01

Next, we differentiated *FTO*^+/+^ and *FTO*^−/−^ hESCs into human mesenchymal progenitor cells (hMPCs) for addressing the effects of FTO deficiency on human adult stem cells (Fig. 1A). Both *FTO*^+/+^ and *FTO*^−/−^ hMPCs expressed typical hMPC surface markers including CD73, CD90, and CD105 (Fig. S1L). We confirmed the loss of FTO protein by western blotting and immunofluorescence staining (Figs. 1I, 1J and S1M). Compared with *FTO*^+/+^ hMPCs, *FTO*^−/−^ hMPCs were able to differentiate into adipocytes, chondrocytes, and osteoblasts, although with a differentiation bias towards the osteoblast fate at the expense of differentiation towards the adipocyte fate (Fig. S1N–P). Strikingly, and different from what we observed in hESCs, self-renewal capacity was impaired in *FTO*^−/−^ hMPCs, as indicated by early-onset growth arrest, restricted clonal expansion, decreased Ki67-positive cells, reduced S phase and prolonged G0/G1 phase (Fig. 1K–N). In addition, FTO ablation led to accelerated senescence, as manifested by elevated senescence-associated β-galactosidase (SA-β-gal) activity, shortened telomere length, downregulated expression of LAP2, HP1α, and H3K9me3, as well as increased nuclear abnormalities and size (Fig. 1O–T). Collectively, these findings indicate that FTO deficiency accelerates hMPC senescence.

We then asked whether the accelerated senescence of *FTO*^−/−^ hMPCs is caused by alterations in the substrate abundance of FTO. Through dot blotting and liquid chromatography assays coupled with tandem mass spectrometry (LC-MS/MS) analysis, we found comparable m^6^A levels in both total RNA and mRNA between *FTO*^+/+^ and *FTO*^−/−^ hESCs (Fig. S2A–C), and between *FTO*^+/+^ and *FTO*^−/−^ hMPCs (Fig. S2D–F). Meanwhile, m^6^A abundance, as detected by fluorometric assays, was also similar between *FTO*^+/+^ and *FTO*^−/−^ hESCs or hMPCs (Fig. S2G and S2H). We then went on to conduct m^6^A-methylated RNA immunoprecipitation sequencing (MeRIP-seq) (Fig. S2I and S2J), and identified similar motif enrichment and distribution patterns of m^6^A methylation between *FTO*^+/+^ and *FTO*^−/−^ hESCs or hMPCs (Fig. 2A–D). There was also no increase in the whole-transcriptome m^6^A peak intensity in FTO-deficient hESCs or hMPCs (Fig. S2K and S2L). Finally, LC-MS/MS analysis of the m^6^A_m_ modification in mRNA and dot blot analysis of the m^1^A modification in transfer RNA (tRNA) revealed no statistical difference between *FTO*^+/+^ and *FTO*^−/−^ hESCs, and between *FTO*^+/+^ and *FTO*^−/−^ hMPCs (Fig. S2M–P). In all, these data demonstrate that FTO depletion exerts minimal impact on global m^6^A, m^6^A_m_ and m^1^A levels in both hESCs and hMPCs, suggesting that FTO may regulate the homeostasis of hMPCs in a demethylation-independent manner.

**Figure 2:**
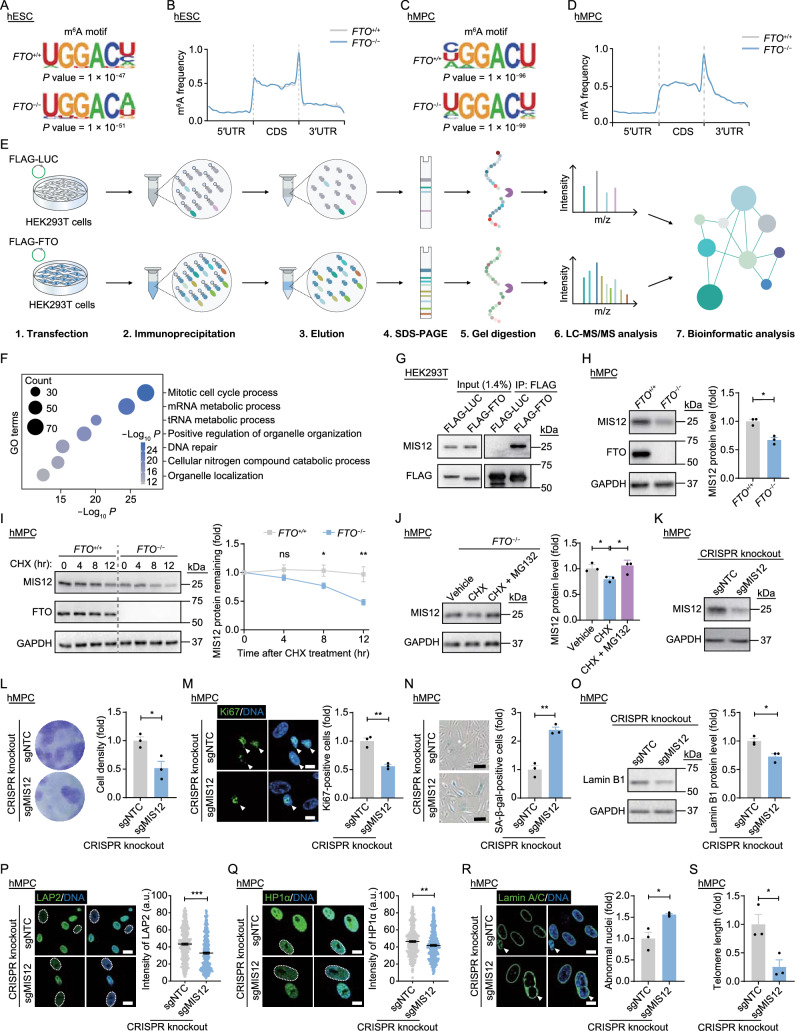
**FTO deficiency impairs MIS12 protein stability to accelerate hMPC senescence in an m**^**6**^**A-independent manner.** (A) Identification of the m^6^A motif by MeRIP-seq analysis in *FTO*^+/+^ and *FTO*^−/−^ hESCs. (B) Distribution of m^6^A peaks along the 5’UTR, CDS, and 3’UTR regions of mRNA from *FTO*^+/+^ and *FTO*^−/−^ hESCs. (C) Identification of the m^6^A motif by MeRIP-seq analysis in *FTO*^+/+^ and *FTO*^−/−^ hMPCs. (D) Distribution of m^6^A peaks along the 5’UTR, CDS, and 3’UTR regions of mRNA from *FTO*^+/+^ and *FTO*^−/−^ hMPCs. (E) A schematic diagram showing the workflow of co-immunoprecipitation (co-IP) assay followed by mass spectrometry analysis. (F) Gene Ontology (GO) enrichment analysis of FTO-interacting proteins identified by mass spectrometry. (G) Co-IP analysis to verify the interaction between MIS12 and FLAG-FTO in HEK293T cells. (H) Western blot analysis of MIS12 and FTO in *FTO*^+/+^ and *FTO*^−/−^ hMPCs. GAPDH was used as the loading control. Data are presented as the means ± SEM. *n* = 3 biological replicates. *, *P* < 0.05. (I) Protein stability analysis of MIS12 in *FTO*^+/+^ and *FTO*^−/−^ hMPCs. Protein levels of MIS12 at indicated time points after treatment with a protein synthesis inhibitor cycloheximide (CHX) were determined by western blotting. GAPDH was used as the loading control. Data are presented as the means ± SEM. *n* = 6 biological replicates. ns, not significant; *, *P* < 0.05; **, *P* < 0.01. (J) Western blot analysis of MIS12 in *FTO*^−/−^ hMPCs with or without the treatment of CHX and MG132. GAPDH was used as the loading control. Data are presented as the means ± SEM. *n* = 3 biological replicates. *, *P* < 0.05. (K) Western blot analysis of MIS12 in control (sgNTC) and *MIS12*-knockout (sgMIS12) hMPCs. GAPDH was used as the loading control. (L) Clonal expansion analysis of control and *MIS12*-knockout hMPCs. Data are presented as the means ± SEM. *n* = 3 biological replicates. *, *P* < 0.05. (M) Immunofluorescence analysis of Ki67 in control and *MIS12*-knockout hMPCs. Scale bars, 20 μm. White arrows indicate Ki67-positive cells. Data are presented as the means ± SEM. *n* = 3 biological replicates. **, *P* < 0.01. (N) SA-β-gal staining of control and *MIS12*-knockout hMPCs. Scale bars, 50 μm. Data are presented as the means ± SEM. *n* = 3 biological replicates. **, *P* < 0.01. (O) Western blot analysis of Lamin B1 in control and *MIS12*-knockout hMPCs. GAPDH was used as the loading control. Data are presented as the means ± SEM. *n* = 3 biological replicates. *, *P* < 0.05. (P) Immunofluorescence analysis of LAP2 in control and *MIS12*-knockout hMPCs. Scale bars, 20 μm. White dashed lines indicate the nuclei with diminished signals of LAP2. Data are presented as the means ± SEM. *n* > 300 cells from three biological replicates. ***, *P* < 0.001. (Q) Immunofluorescence analysis of HP1α in *FTO*^+/+^ and *FTO*^−/−^ hMPCs. Scale bars, 20 μm. White dashed lines indicate the nuclei with diminished signals of HP1α. Data are presented as the means ± SEM. *n* > 300 cells from three biological replicates. **, *P* < 0.01. (R) Immunofluorescence analysis of Lamin A/C in *FTO*^+/+^ and *FTO*^−/−^ hMPCs. Scale bars, 20 μm. White arrows indicate abnormal nuclei. Data are presented as the means ± SEM. *n* = 3 biological replicates. *, *P* < 0.05. (S) Telomere length analysis of control and *MIS12*-knockout hMPCs. Data are presented as the means ± SEM. *n* = 3 biological replicates. *, *P* < 0.05

Previous studies reported that FTO interacts with downstream proteins to enhance their biological activity (Tao et al., 2020). To identify such a potential mechanism for FTO in regulating hMPC senescence, we ectopically expressed FLAG-tagged FTO proteins in HEK293T cells and conducted co-immunoprecipitation (co-IP) assay with an anti-FLAG antibody, followed by LC-MS/MS analysis (Fig. 2E and Table S1). We identified a panel of potential FTO-interacting proteins (Table S1) that were found to be mostly enriched for “mitotic cell cycle process” through Gene Ontology (GO) enrichment analysis (Fig. 2F). Among these was MIS12 (Fig. S2Q and S2R), a key regulator of cell cycle distribution that we had reported in the regulation of hMPC senescence (Wu et al., 2020). Here, we validated the interaction between FTO and MIS12 through co-IP assay (Fig. 2G). Then, we asked whether MIS12 protein levels might be influenced by FTO depletion. Indeed, western blot analysis revealed a marked decrease of MIS12 expression in *FTO*^−/−^ hMPCs rather than in *FTO*^−/−^ hESCs (Figs. 2H and S2S), indicating a cell-type-specific regulation. When we analyzed the mRNA and m^6^A levels of *MIS12*, we found no remarkable differences between *FTO*^+/+^ and *FTO*^−/−^ hMPCs, as revealed by the MeRIP-seq and RT-qPCR results (Fig. S2T–W). These data indicate that FTO regulates the expression of MIS12 protein in an m^6^A-independent manner. We hence speculated that FTO might regulate hMPC senescence by stabilizing MIS12 protein. Indeed, in protein stability assay, we detected an increased MIS12 degradation upon FTO deficiency (Fig. 2I). To confirm the pathway by which FTO ablation facilitated the protein degradation of MIS12, we treated *FTO*^−/−^ hMPCs with MG132 (a proteasome inhibitor) and bafilomycin A1 (BFA1, an autophagy inhibitor), respectively. As shown by the western blotting results, treatment with MG132 instead of BFA1 rescued the MIS12 protein abundance in *FTO*^−/−^ hMPCs to a similar level with that in *FTO*^+/+^ hMPCs (Fig. S2X and S2Y). Furthermore, treatment with MG132 restored the MIS12 protein levels that were further decreased upon cycloheximide (CHX) addition in *FTO*^−/−^ hMPCs (Fig. 2J), indicating that FTO ablation accelerates MIS12 degradation via the proteasome pathway.

To further dissect the role of MIS12 in regulating hMPC senescence, we next ablated MIS12 in WT hMPCs using a lentivirus-mediated CRISPR/Cas9 knockout system (Figs. 2K and S2Z). We found that depletion of MIS12 accelerated hMPC senescence, as evidenced by compromised clonal expansion ability, reduced Ki67-positive cells, increased SA-β-gal activity, downregulated expression of Lamin B1, LAP2, and HP1α, and more abnormal nuclei, as well as shortened telomere length (Fig. 2L–S). Accordingly, these findings suggest that the presence of MIS12 counteracts acquisition of hMPC senescence.

In conclusion, we here used CRISPR/Cas9-based techniques to generate FTO-deficient hESCs and hMPCs, an approach through which we gained insights into the regulatory role of FTO and molecular mechanisms in homeostatic maintenance of human stem cells. As demonstrated by our data, FTO was dispensable for the maintenance of hESC pluripotency, consistent with a recent related study (Wei et al., 2021). In contrast, we demonstrated that ablation of FTO accelerated hMPC senescence, as manifested by decreased cell proliferation, impaired cell cycle and increased SA-β-gal activity. In line with our observations in FTO-deficient hMPCs, previous studies have unveiled that inhibition of FTO led to impeded cell cycle progression and compromised cell proliferation in myoblasts and a variety of cancer cells (Huang et al., 2020; Deng et al., 2021). Moreover, FTO knockdown in human ovarian granulosa cells also resulted in accelerated senescence (Jiang et al., 2021). Collectively, these reports and our study demonstrate cell-type-specific roles of FTO.

Cell cycle arrest is a common feature of cellular senescence (Wu et al., 2020). Consistently, we also detected aberrant cell cycle progress in FTO-deficient hMPCs. Furthermore, LC-MS/MS analysis identified a panel of cell cycle-related FTO-interacting proteins, indicating a potential role of these cell cycle factors in regulating hMPC senescence upon FTO deficiency. Subsequently, we noticed MIS12, a cell cycle regulator that interacts with DSN1, NSL1, and PMF1, to form the kinetochore complex, thus promoting chromosome segregation during mitosis and being involved in the regulation of cell proliferation (Abe-Kanoh et al., 2019). Indeed, our previous study has demonstrated that downregulation of MIS12 due to m^6^A reduction decreased cell proliferation and accelerated senescence in hMPCs (Wu et al., 2020). Coincidentally, we also observed a decrease in the protein level of MIS12 in FTO-deficient hMPCs, and that knockout of MIS12 led to compromised self-renewal and premature senescence in WT hMPCs, mimicking majority of the senescent phenotypes caused by FTO deficiency. Discordantly, we did not detect any visible alterations in the amounts of both m^6^A modification and mRNA level of *MIS12* upon FTO depletion, indicating an m^6^A-independent way by which FTO regulates the expression of MIS12. Apart from MIS12, NSL1, another component of the kinetochore complex (Abe-Kanoh et al., 2019), was also identified as a candidate protein interacting with FTO (Fig. S2Q and S2R; Table S1), supporting the implication of cell cycle regulators in FTO-mediated biological process. Our further results demonstrated that FTO deficiency facilitated the degradation of MIS12 through the proteasome-mediated pathway. Although previous work supported that MIS12 can be degraded via the ubiquitin-proteasome system (Abe-Kanoh et al., 2019), how FTO protects MIS12 from degradation in hMPCs awaits further investigations. To be noted, though we demonstrated MIS12 as a downstream factor of FTO in regulating hMPC senescence, we could not rule out the possibility that there exist some other potential partners also involved in this process. For example, RB1 and CDKN2A, two well-known senescence regulators, were also identified as potential FTO-interacting proteins (Fig. S2Q and Table S1). Whether RB1 or CDKN2A partially contributes to hMPC senescence caused by FTO deficiency needs to be explored.

Collectively, our study disclosed an uncharacterized role of FTO in maintaining the protein stability of MIS12, independent of its m^6^A demethylation activity, and in counteracting hMPC senescence. These new findings further increase the complexity of FTO in the regulation of cellular physiology. Yet, more efforts need to be integrated to figure out the possible explanation for the globally unchanged m^6^A abundance in FTO-deficient human stem cells.

## Supplementary Information

Below is the link to the electronic supplementary material.

Supplementary file1 (PDF 2229 kb)

Supplementary file2 (XLSX 119 kb)

Supplementary file3 (XLSX 16 kb)

## References

[CR1] Abe-Kanoh N, Kunisue N, Myojin T, Chino A, Munemasa S, Murata Y, Satoh A, Moriya H, Nakamura Y (2019). Yeast screening system reveals the inhibitory mechanism of cancer cell proliferation by benzyl isothiocyanate through down-regulation of Mis12. Sci Rep.

[CR2] Bi C, Wang L, Yuan B, Zhou X, Li Y, Wang S, Pang Y, Gao X, Huang Y, Li M (2020). Long-read individual-molecule sequencing reveals CRISPR-induced genetic heterogeneity in human ESCs. Genome Biol.

[CR3] Deng K, Zhang Z, Ren C, Liang Y, Gao X, Fan Y, Wang F (2021). FTO regulates myoblast proliferation by controlling CCND1 expression in an m(6)A-YTHDF2-dependent manner. Exp Cell Res.

[CR4] Fischer J, Koch L, Emmerling C, Vierkotten J, Peters T, Brüning JC, Rüther U (2009). Inactivation of the Fto gene protects from obesity. Nature.

[CR5] Huang H, Weng H, Chen J (2020). m(6)A modification in coding and non-coding RNAs: roles and therapeutic implications in cancer. Cancer Cell.

[CR6] Jia G, Fu Y, Zhao X, Dai Q, Zheng G, Yang Y, Yi C, Lindahl T, Pan T, Yang YG (2011). N6-methyladenosine in nuclear RNA is a major substrate of the obesity-associated FTO. Nat Chem Biol.

[CR7] Jiang ZX, Wang YN, Li ZY, Dai ZH, He Y, Chu K, Gu JY, Ji YX, Sun NX, Yang F (2021). The m6A mRNA demethylase FTO in granulosa cells retards FOS-dependent ovarian aging. Cell Death Dis.

[CR8] Li L, Zang L, Zhang F, Chen J, Shen H, Shu L, Liang F, Feng C, Chen D, Tao H (2017). Fat mass and obesity-associated (FTO) protein regulates adult neurogenesis. Hum Mol Genet.

[CR9] Li W, Zou Z, Cai Y, Yang K, Wang S, Liu Z, Geng L, Chu Q, Ji Z, Chan P (2022). Low-dose chloroquine treatment extends the lifespan of aged rats. Protein Cell.

[CR10] Shan H, Geng L, Jiang X, Song M, Wang J, Liu Z, Zhuo X, Wu Z, Hu J, Ji Z (2021). Large-scale chemical screen identifies Gallic acid as a geroprotector for human stem cells. Protein Cell.

[CR11] Sun H, Li K, Zhang X, Liu J, Zhang M, Meng H, Yi C (2021). m(6)Am-seq reveals the dynamic m(6)Am methylation in the human transcriptome. Nat Commun.

[CR12] Tao B, Huang X, Shi J, Liu J, Li S, Xu C, Zhong J, Wan L, Feng B, Li B (2020). FTO interacts with FOXO3a to enhance its transcriptional activity and inhibits aggression in gliomas. Signal Transduct Target Ther.

[CR14] Wei J, Liu F, Lu Z, Fei Q, Ai Y, He PC, Shi H, Cui X, Su R, Klungland A (2018). Differential m(6)A, m(6)A(m), and m(1)A demethylation mediated by FTO in the cell nucleus and cytoplasm. Mol Cell.

[CR13] Wei C, Luo Q, Wang B, Long Y, Zhang M, Shan W, Yu X, Xu Y, Qian P, Huang H (2021). Generation of a FTO gene knockout human embryonic stem cell line using CRISPR/Cas9 editing. Stem Cell Res.

[CR15] Wu Z, Shi Y, Lu M, Song M, Yu Z, Wang J, Wang S, Ren J, Yang YG, Liu GH (2020). METTL3 counteracts premature aging via m6A-dependent stabilization of MIS12 mRNA. Nucleic Acids Res.

