# Supplementary material for: FTO stabilizes MIS12 and counteracts senescence
Source: Protein Cell. 2022 Apr 6;13(12):954–60. doi: 10.1007/s13238-022-00914-6 (PMC9243202; doi:10.1007/s13238-022-00914-6)
Supplement: Supplementary file 1 — Supplementary file1 (PDF 2229 kb) [file 13238_2022_914_MOESM1_ESM.pdf]

Supplemental Materials

Materials and methods

Cell culture

Wild-type (WT, *FTO*$^{+/+}$) hESCs (Line H9, WiCell Research) and *FTO*$^{-/-}$ hESCs were cultured on mitomycin C-inactivated mouse embryonic fibroblast (MEFs) in hESC culture medium containing 80% DMEM/F12 basal medium (Thermo Fisher Scientific), 20% Knockout Serum Replacement (Thermo Fisher Scientific), 0.1 mM non-essential amino acids (NEAA, Thermo Fisher Scientific), 2 mM GlutaMAX (Thermo Fisher Scientific), 1% penicillin/streptomycin (Thermo Fisher Scientific), 55 μM β-mercaptoethanol (Thermo Fisher Scientific), and 10 ng/mL bFGF (Joint Protein Central). hESCs were also cultured on Matrigel (BD Biosciences)-coated plates in mTeSR medium (STEMCELL Technologies). hMPCs were cultured in hMPC culture medium containing 90% MEMα with GlutaMAX basal medium (Thermo Fisher Scientific), 10% fetal bovine serum (Gibco), 1% penicillin/streptomycin, 0.1 mM NEAA and 1 ng/mL bFGF.

Generation of *FTO*$^{-/-}$ hESCs by CRISPR/Cas9-based strategy

FTO$^{-/-}$ hESCs were generated by CRISPR/Cas9-based gene editing as previously described (Abakir et al., 2020; Li et al., 2020) with some modifications. Briefly, sgRNA targeting the first exon of *FTO* gene was used as previously described (Xiang et al., 2017), and cloned into pCAG-mCherry-sgRNA vector (Addgene, #87110). Then, the reconstructed pCAG-mCherry-sgRNA vector together with pCAG-1BPNLS-Cas9-1BPNLS-2AGFP vector (Addgene, #87109) was electroporated into *FTO*$^{+/+}$ hESCs using a 4D-Nucleofector (Lonza). After electroporation, cells were seeded on Matrigel-coated plates and cultured in mTeSR medium supplemented with ROCK inhibitor Y-27632 (Tocris) for 48 hr. mCherry/GFP-double-positive cells were sorted using a flow cytometer (BD, Aria II) and cultured on mitomycin C-inactivated MEFs with hESC culture medium. Emerging hESC clones were processed for genomic DNA extraction,

PCR amplification and DNA sequencing. The potential off-target sites were predicted using the website http://crispr.mit.edu. sgRNA sequences for gene editing and primers for clone identification are listed in Table S2.

Generation of hMPCs by directed differentiation from hESCs

FTO$^{+/+}$ and *FTO*$^{-/-}$ hMPCs were differentiated from corresponding hESCs as previously described (Shan et al., 2021; Wang et al., 2021a). Briefly, *FTO*$^{+/+}$ and *FTO*$^{-/-}$ hESCs were dissociated into embryonic bodies and seeded on Matrigel-coated plates in hMPC differentiation medium containing 90% MEMα with GlutaMAX basal medium, 10% fetal bovine serum, 0.1 mM NEAA, 1% penicillin/streptomycin, 10 ng/mL bFGF and 5 ng/mL TGFβ (Humanzyme). Around 10 days later, fibroblast-like cells were collected and transferred onto Gelatin-coated plates for further culture with hMPC culture medium. Subsequently, CD73, CD90, CD105 triple-positive cells (hMPCs) were sorted using a flow cytometer and cultured in hMPC culture medium. Antibodies used for flow cytometry analysis include anti-CD73-PE (BD Biosciences, 550257), anti-CD90-FITC (BD Biosciences, 555595), and anti-CD105-APC (BioLegend, 800508). To evaluate the triple-lineage differentiation potential of hESC-derived hMPCs toward adipocytes, chondrocytes and osteoblasts, Oil Red O (Sigma) staining, Toluidine Blue O (Sigma) staining and von Kossa (GENMED) staining were conducted, respectively.

Analysis of DNA methylation at *OCT4* promoter region

The DNA methylation state at *OCT4* promoter region in hESCs was determined as previously reported (Ling et al., 2019). In brief, genomic DNAs were isolated using a DNA Extraction Kit (TIANGEN) and subjected to bisulfite treatment using an EZ DNA Methylation Kit (Zymo Research). The bisulfite treated genomic DNAs were processed for PCR amplification using LA Taq DNA Polymerase Hot-Start Version (TaKaRa) to acquire DNA fragments at *OCT4* promoter region. Then, the PCR products were purified and cloned into the pMD20 T vector (TaKaRa). Six clones of each hESC line were picked out and sequenced using the universal primer M13. Primers used for DNA methylation analysis are listed in Table S2.

Immunofluorescence staining

For immunofluorescence staining, cells seeded on coverslips (Thermo Fisher Scientific) were fixed with 4% paraformaldehyde for 15 min, and permeabilized in 0.2% Triton X-100 for 10 min at room temperature. After permeabilization, cells were blocked with 10% donkey serum in PBS for 1 hr at room temperature, and then incubated with the primary antibody at 4°C overnight. After washing with PBS for three times, cells were incubated with fluorescence-labeled secondary antibody at room temperature for 1 hr. Nuclear DNA was stained with Hoechst 33342 (Thermo Fisher Scientific). Subsequently, the coverslips were mounted with mounting medium (Vector Labs). Images were captured with a Leica SP5 confocal system. ImageJ was used for immunofluorescence intensity measurement.

Antibodies used for immunofluorescence staining include anti-FTO (Abcam, ab126605), anti-OCT4 (Santa Cruz Biotechnology, sc-5279), anti-SOX2 (R&D, MAB2018), anti-NANOG (Abcam, ab109250), anti-Ki67 (ZSGB-BIO, ZM-0166), anti-LAP2 (BD Biotechnology, 611000), anti-HP1α (Cell Signaling Technology, 2616), anti-H3K9me3 (Abcam, ab8898), anti-Lamin A/C (Santa Cruz Biotechnology, sc-37628), Alexa 488 donkey anti-mouse IgG (Thermo Fisher Scientific, A21202), Alexa 488 donkey anti-rabbit IgG (Thermo Fisher Scientific, A21206), Alexa 568 donkey anti-rabbit IgG (Thermo Fisher Scientific, A10042), and Alexa 647 donkey anti-goat IgG (Thermo Fisher Scientific, A21447).

Lentiviral CRISPR/Cas9-mediated *MIS12* knockout

Lentiviral CRISPR/Cas9-mediated gene knock out was performed as previously described (Wang et al., 2021b). In brief, the sgRNA targeting *MIS12* (sgMIS12) was cloned into lenti-CRISPRv2 vector (Addgene, #52961) containing an hSpCas9 expression cassette. Then, the reconstructed lentiviral vectors together with psPAX2 (Addgene, #12260) and pMD2G (Addgene, #12259) were co-transfected into HEK293T cells for lentivirus production. Lentiviruses carrying sgMIS12 or control sgRNA (non-targeting control, sgNTC) were transduced into *FTO*$^{+/+}$ hMPCs. At 48 hr post-transfection, cells were

treated with 1 μg/mL puromycin (Thermo Fisher Scientific) for around 5 days. Subsequently, the knockout efficiency of MIS12 was examined by western blot analysis and further phenotypic analysis was conducted after two passages.

SA-β-gal staining

SA-β-gal staining of hMPCs were performed as previously described (Geng et al., 2019; Lei et al., 2021). Briefly, cells were washed with PBS for three times, fixed with fixation solution containing 2% formaldehyde and 0.2% glutaraldehyde in PBS for 4 min at room temperature, and then stained with staining solution at 37°C overnight. Then, images were obtained using a microscope digital camera (Olympus). ImageJ was used to calculate the percentage of SA-β-gal-positive cells.

Clonal expansion assay

Clonal expansion assay in hMPCs was performed as previously described (Geng et al., 2019; Wu et al., 2018). In brief, 2,000 hMPCs per well were seeded in a 12-well plate coated with 0.1% Gelatin, and cultured to almost confluence. Then, cells were fixed with 4% paraformaldehyde for 30 min at room temperature. After washing with PBS for three times, the fixed cells were stained with 0.2% crystal violet for 1 hr at room temperature. Subsequently, images were captured using an optical scanner (Hewlett-Packard) and cell density of each well was quantified.

Co-IP assay

Co-IP assay was performed as previously reported (Deng et al., 2019; Liang et al., 2021). In brief, HEK293T cells transfected with vectors expressing FLAG-LUC or FLAG-FTO were harvested, and processed for lysis in CHAPS lysis buffer containing 120 mM NaCl, 0.3% CHAPS, 40 mM HEPES, 1 mM EDTA, and complete protease inhibitor cocktail (Roche). After cell lysis, a centrifugation at 12, 000 *g* for 30 min was conducted to collect the supernatant, which was then subjected to incubation with anti-FLAG Affinity Gel (Sigma, A2220) at 4°C overnight. FTO-interacting protein complexes were obtained by

competitive elution using FLAG peptides, and processed for western blotting or LC-MS/MS analysis.

LC-MS/MS analysis for identifying protein-protein interactions

LC-MS/MS analysis was performed for identifying FTO-interacting proteins as previously reported (Deng et al., 2019; Liang et al., 2021). In brief, isolated proteins from the co-IP assay were separated using SDS-PAGE gel and then stained with Coomassie Brilliant Blue. Protein bands were excised from the SDS-PAGE gel and subjected to in-gel digestion with sequencing-grade Trypsin (Worthington). Peptides extracted from the gel were processed for mass spectrometry analysis using a nanoLC-Q Exactive Mass Spectrometer (Thermo Fisher Scientific). Data analysis was conducted using MaxQuant (Version 1.3) and Proteome Discoverer (Version 1.4). False discovery rate (FDR) less than 1% was set as the threshold of the peptide confidence parameter. Candidate proteins that were only present in the FLAG-FTO group were considered as high-confidence FTO-interacting proteins. For functional enrichment analysis of FTO-interacting proteins, Gene Ontology (GO) analysis of interested proteins was performed using metascape (http://metascape.org) (Zhou et al., 2019). The functional terms with $P < 0.05$ were set to be statistically significant. FTO-interacting proteins are listed in Table S1.

Western blotting

Briefly, proteins were extracted from hESCs or hMPCs in 1 × SDS lysis buffer (62.5 mM Tris-HCl pH = 6.8, 2% SDS) and quantified using a BCA Protein Quantification Kit (Dingguochangsheng). 20 μg protein per sample was subjected to SDS-PAGE electrophoresis and electrotransferred to PVDF membrane (Millipore). After electrotransferation, the membrane was blocked with 5% non-fat milk, and incubated with antibodies at 4°C overnight and then with HRP-conjugated secondary antibodies at room temperature for 1 hr. Subsequently, imaging was performed using a ChemiDoc XRS+ system (Bio-Rad) and intensity of target protein bands was determined using ImageJ.

Antibodies used for western blotting include anti-FTO (Abcam, ab126605), anti-MIS12 (Abcam, ab70843), anti-GAPDH (Santa Cruz Biotechnology, sc-365062), anti-HP1α (Cell Signaling Technology, 2616), anti-Lamin B1 (Abcam, ab16048), anti-FLAG (Sigma, F1804), anti-LAP2 (BD Bioscience, 611000), HRP-conjugated goat anti-mouse IgG (ZB-2305), and HRP-conjugated goat anti-rabbit IgG (ZB-2301).

RT-qPCR

Total RNA was extracted using TRIzol reagent (Thermo Fisher Scientific). cDNA was generated using the GoScript Reverse Transcription System (Promega), and then subjected to RT-qPCR analysis using SYBR Green Master Mix (TOYOBO) in a CFX-384 Real-Time PCR system (Biosystem). Primers used for RT-qPCR analysis are listed in Table S2.

Detection of telomere length

Telomere length detection was performed as previously described (Hu et al., 2020; Zhang et al., 2019a). In brief, genomic DNA was extracted from hMPCs using a DNA Extraction Kit. Then, the SYBR Green Master Mix was mixed with DNA samples and qPCR analysis was conducted using a CFX-384 Real-Time PCR system. Primers used for telomere length analysis are listed in Table S2.

Dot blotting

m^6A and m^1A dot blotting were performed as previously described (Wei et al., 2018; Wu et al., 2020). In brief, total RNA was extracted using TRIzol reagent. mRNA was isolated using a Dynabeads mRNA Purification Kit (Thermo Fisher Scientific). tRNA was separated from total RNA by gels containing 15% polyacrylamide and 8 M urea (Lin et al., 2013) and recovered using ZR small-RNA PAGE Recovery Kit (Zymo Research, R1070). 200 ng of total RNA, mRNA or tRNA were loaded on nylon membrane and processed for UV crosslinking. Subsequently, the membranes were blocked with 5% non-fat milk, and incubated with antibody at 4°C overnight. After incubation with HRP-conjugated secondary antibody at room temperature for 1 hr, a ChemiDoc

XRS+ system was used for imaging. The dot blot intensity was determined using ImageJ and methylene blue (MB) staining was used as loading control.

Antibodies used for dot blotting include anti-m^6A (Synaptic systems, 202003), anti-m^1A (Medical & Biological Laboratories, D345-3), HRP-conjugated goat anti-mouse IgG (ZB-2305), and HRP-conjugated goat anti-rabbit IgG (ZB-2301).

Detection of m^6A level by fluorometric assay

Fluorometric assay for detecting the overall m^6A level was performed using the EpiQuik m^6A RNA Methylation Quantification Kit (Fluorometric) (Epigentek, P-9008-96) according to the manufacturer’s instructions. In brief, 200 ng total RNA or mRNA together with negative and positive controls were added into the designated wells. After incubation with capture and detection antibodies, the overall m^6A abundance was quantified fluorometrically with a fluorescence spectrophotometer (BioTek).

Measurement of m^6A and m^6A_m abundance using LC-MS/MS analysis

Analysis of m^6A and m^6A_m abundance by LC-MS/MS was performed as previously described (Liu et al., 2020; Sendinc et al., 2019; Zhang et al., 2019b). 250 ng mRNA was de-capped by Cell-Clip enzyme (Cellscript) using 0.5 U enzyme at 37°C for 1 hr. Then, the de-capped mRNA was digested with 1 U nuclease P1 (Wako USA, 145-08221, Lot# CAJ3980) in 10 mM NH_4OAc (pH = 5.3) at 42°C for 2 hr. And then mono nucleic acid from last step was digested with rSAP (NEB) at 37°C for 1 hr. The samples were filtered by 0.22-μm filter (Pall Corporation) and 10 μL sample was injected into a triple-quadrupole mass spectrometer (AB SCIEX QTRAP 6500+) with C18 column for further analysis. The positive ion multiple reaction-monitoring (MRM) mode was used to detect modification base level. The nucleoside to base ion mass transition were used to qualify m^6A and m^6A_m levels: 268.0 to 136.0 for A, 282.0 to 105.1 for m^6A, and 296.0 to 105.1 for m^6A_m. Commercial A (Berry & Associates, PR3005), m^6A (Berry & Associates, PR3732), and m^6A_m (Berry & Associates, PR3733) were used as standard samples.

Protein stability analysis

Protein stability analysis was performed as previously described (Jeong et al., 2019). Briefly, cells cultured to 80% confluence were treated with 20 μg/mL cycloheximide (CHX, Sigma), and pellets were collected at 0, 4, 8, 12 hr post-treatment. Then, cell pellets were processed for western blotting. To test whether the degradation of MIS12 in hMPC depends on proteasome or autophagy-mediated pathway, *FTO*$^{-/-}$ hMPCs were treated with 20 μM MG132 (Sigma) or 50 nM BFA1 (Sigma) for 12 hr, and then collected for western blotting.

CNV analysis

CNV analysis was performed as previously reported (Bi et al., 2020). In brief, the genomic DNAs of *FTO*$^{+/+}$ and *FTO*$^{-/-}$ hESCs were extracted using a DNeasy Blood & Tissue Kit (QIAGEN). Then, DNA quality control, library preparation and high-throughput sequencing on Illumina HiSeq X Ten platforms were performed by Novogene Bioinformatics Technology Co. Ltd. Bioinformatic analysis was conducted using the published R packages. Briefly, raw reads were trimmed with TrimGalore, aligned to the human hg19 genome reference, and counted for each 500-Kb window using readCounter in hmmcopy_utils (https://github.com/shahcompbio/hmmcopy_utils). Correction of the copy number, GC content and mappability was performed using the R/Bioconductor package HMMcopy (Version 1.26.0).

MeRIP assay

MeRIP assay was performed as previously reported (Wu et al., 2020). Total RNA was extracted from cells by TRIzol reagent. And mRNA was isolated by the Dynabeads mRNA Purification Kit (Thermo Fisher Scientific, 61006). After digestion by DNase I, the purified mRNA was fragmented to around 100-nt with RNA Fragmentation Reagent (Thermo Fisher Scientific, AM8740) at 94°C for 45 s. To terminate the reaction, the stop reagent (Thermo Fisher Scientific, AM8740) was added. And then the fragmented RNA was precipitated using pure ethanol. To capture the m^{6}A-tagged fragmented RNA, the m^{6}A antibody

(Synaptic systems) was pre-incubated with Dynabeads Protein A (Thermo Fisher Scientific, 10013D) in IPP buffer (150 mM NaCl, 0.1% NP-40, 10 mM Tris-HCl pH = 7.4) at room temperature for 1 hr. Subsequently, the fragmented RNA was heated at 75°C for 5 min, followed by chilling on ice, and then mixed with the antibody-beads complex for further incubation at 4°C for 4 hr. After adequately washing, the bounded RNA was obtained by competitively binding with m^6A-modified adenosine (Abcam, ab145715), extracted by phenol-chloroform (Thermo Fisher Scientific, AM9730), and washed by pure ethanol. The collected RNA was used for RT-qPCR analysis and library construction. For MeRIP-seq library construction, the immunoprecipitated RNA was processed using a SMARTer smRNA-Seq Kit (TaKaRa, 635030), with a small amount of fragmented input RNA for RNA-seq library construction using a KAPA Stranded RNA-Seq Library Preparation Kit (KAPA, KK8401). Finally, high-throughput sequencing was performed on Illumina HiSeq X Ten platforms by Novogene Bioinformatics Technology Co., Ltd.

Analysis of MeRIP-seq and RNA-seq

For general read pre-processing of MeRIP-seq, there are two biological replicates prepared for each sample. Adaptors and low-quality reads were trimmed off for all raw reads using the Trimmomatic (Version 0.36) (Bolger et al., 2014). The remaining reads were aligned to the human reference genome (hg19) using HISAT2 software (Version 2.1.0) (Kim et al., 2019). Only uniquely mapped reads with mapping quality score ≥ 20 were kept for the subsequent analysis for each sample.

For peak calling and motif enrichment analysis of MeRIP-seq, MACS2 peak-calling software (Version 2.1.2) was used to call the m^6A peaks with the default options excluding '–nomodel, –keepdup all' (Zhang et al., 2008). The high-confidence peaks were obtained using a stringent cutoff threshold *P*-value of 1×10^{-5}. BEDTools 'IntersectBed' (Version 2.25.0) was applied for peak annotation based on UCSC gene annotation information (Quinlan and Hall, 2010). Motif enrichment was analyzed by HOMER (Version 2.1.2) with a motif length of 6-nucleotide selection (Heinz et al., 2010). Background regions were

generated by shuffling peaks along the total mRNA using the shuffleBed tool from the BEDtools software.

For RNA-seq analysis, reads mapped to hg19 reference were used to calculate the transcripts per million (TPM) of each gene by StringTie (Version 1.3.6) (Pertea et al., 2015).

Statistical analysis

Statistical analyses in this study are performed using Two-tailed Student's *t*-test with Graph-Pad Prism. Results are shown as the means ± SEM. P-value < 0.05 (*), P-value < 0.01 (**), and P-value < 0.001 (***) are considered as statistically significant.

Data availability

MeRIP-seq data generated in this study have been deposited in the Genome Sequence Archive (GSA) in the National Genomics Data Center, Beijing Institute of Genomics (China National Center for Bioinformation) of the Chinese Academy of Sciences, under accession number HRA001575 that are publicly accessible at http://bigd.big.ac.cn/gsa-human. Mass spectrometry proteomics data have been deposited in the ProteomeXchange Consortium via the PRIDE partner repository with the dataset identifier PXD029892.

References

Abakir, A., Giles, T.C., Cristini, A., Foster, J.M., Dai, N., Starczak, M., Rubio-Roldan, A., Li, M., Eleftheriou, M., Crutchley, J.*, et al.* (2020). N(6)-methyladenosine regulates the stability of RNA:DNA hybrids in human cells. Nat Genet *52*, 48-55.

Bi, S., Liu, Z., Wu, Z., Wang, Z., Liu, X., Wang, S., Ren, J., Yao, Y., Zhang, W., Song, M.*, et al.* (2020). SIRT7 antagonizes human stem cell aging as a heterochromatin stabilizer. Protein Cell *11*, 483-504.

Bolger, A.M., Lohse, M., and Usadel, B. (2014). Trimmomatic: a flexible trimmer for Illumina sequence data. Bioinformatics *30*, 2114-2120.

Deng, L., Ren, R., Liu, Z., Song, M., Li, J., Wu, Z., Ren, X., Fu, L., Li, W., Zhang, W.*, et al.* (2019). Stabilizing heterochromatin by DGCR8 alleviates senescence and osteoarthritis. Nat Commun *10*, 3329.

Geng, L., Liu, Z., Zhang, W., Li, W., Wu, Z., Wang, W., Ren, R., Su, Y., Wang, P., Sun, L.*, et al.* (2019). Chemical screen identifies a geroprotective role of quercetin in premature aging. Protein Cell *10*, 417-435.

Heinz, S., Benner, C., Spann, N., Bertolino, E., Lin, Y.C., Laslo, P., Cheng, J.X., Murre, C., Singh, H., and Glass, C.K. (2010). Simple combinations of lineage-determining transcription factors prime cis-regulatory elements required for macrophage and B cell identities. Mol Cell *38*, 576-589.

Hu, H., Ji, Q., Song, M., Ren, J., Liu, Z., Wang, Z., Liu, X., Yan, K., Hu, J., Jing, Y.*, et al.* (2020). ZKSCAN3 counteracts cellular senescence by stabilizing heterochromatin. Nucleic Acids Res *48*, 6001-6018.

Jeong, W.J., Park, J.C., Kim, W.S., Ro, E.J., Jeon, S.H., Lee, S.K., Park, Y.N., Min, D.S., and Choi, K.Y. (2019). WDR76 is a RAS binding protein that functions as a tumor suppressor via RAS degradation. Nat Commun *10*, 295.

Kim, D., Paggi, J.M., Park, C., Bennett, C., and Salzberg, S.L. (2019). Graph-based genome alignment and genotyping with HISAT2 and HISAT-genotype. Nat Biotechnol *37*, 907-915.

Lei, J., Wang, S., Kang, W., Chu, Q., Liu, Z., Sun, L., Ji, Y., Esteban, C.R., Yao, Y., Belmonte, J.C.I.*, et al.* (2021). FOXO3-engineered human mesenchymal progenitor cells efficiently promote cardiac repair after myocardial infarction. Protein Cell *12*, 145-151.

Li, H., Wu, Z., Liu, X., Zhang, S., Ji, Q., Jiang, X., Liu, Z., Wang, S., Qu, J., Zhang, W.*, et al.* (2020). ALKBH1 deficiency leads to loss of homeostasis in human diploid somatic cells. Protein Cell *11*, 688-695.

Liang, C., Liu, Z., Song, M., Li, W., Wu, Z., Wang, Z., Wang, Q., Wang, S., Yan, K., Sun, L.*, et al.* (2021). Stabilization of heterochromatin by CLOCK promotes stem cell rejuvenation and cartilage regeneration. Cell Res *31*, 187-205.

Lin, J., Lu, J., Feng, Y., Sun, M., and Ye, K. (2013). An RNA-binding complex involved in ribosome biogenesis contains a protein with homology to tRNA CCA-adding enzyme. PLoS Biol *11*, e1001669.

Ling, C., Liu, Z., Song, M., Zhang, W., Wang, S., Liu, X., Ma, S., Sun, S., Fu, L., Chu, Q.*, et al.* (2019). Modeling CADASIL vascular pathologies with patient-derived induced pluripotent stem cells. Protein Cell *10*, 249-271.

Liu, J., Li, K., Cai, J., Zhang, M., Zhang, X., Xiong, X., Meng, H., Xu, X., Huang, Z., Peng, J.*, et al.* (2020). Landscape and Regulation of m(6)A and m(6)Am Methylome across Human and Mouse Tissues. Mol Cell *77*, 426-440 e426.

Pertea, M., Pertea, G.M., Antonescu, C.M., Chang, T.C., Mendell, J.T., and Salzberg, S.L. (2015). StringTie enables improved reconstruction of a transcriptome from RNA-seq reads. Nat Biotechnol *33*, 290-295.

Quinlan, A.R., and Hall, I.M. (2010). BEDTools: a flexible suite of utilities for comparing genomic features. Bioinformatics *26*, 841-842.

Sendinc, E., Valle-Garcia, D., Dhall, A., Chen, H., Henriques, T., Navarrete-Perea, J., Sheng, W., Gygi, S.P., Adelman, K., and Shi, Y. (2019). PCIF1 Catalyzes m6Am mRNA Methylation to Regulate Gene Expression. Mol Cell *75*, 620-630.e629.

Shan, H., Geng, L., Jiang, X., Song, M., Wang, J., Liu, Z., Zhuo, X., Wu, Z., Hu, J., Ji, Z.*, et al.* (2021). Large-scale chemical screen identifies Gallic acid as a geroprotector for human stem cells. Protein Cell.

Wang, S., Cheng, F., Ji, Q., Song, M., Wu, Z., Zhang, Y., Ji, Z., Feng, H., Belmonte, J.C.I., Zhou, Q.*, et al.* (2021a). Hyperthermia differentially affects specific human stem cells and their differentiated derivatives. Protein Cell.

Wang, W., Zheng, Y., Sun, S., Li, W., Song, M., Ji, Q., Wu, Z., Liu, Z., Fan, Y., Liu, F.*, et al.* (2021b). A genome-wide CRISPR-based screen identifies KAT7 as a driver of cellular senescence. Sci Transl Med *13*.

Wei, J., Liu, F., Lu, Z., Fei, Q., Ai, Y., He, P.C., Shi, H., Cui, X., Su, R., Klungland, A.*, et al.* (2018). Differential m(6)A, m(6)A(m), and m(1)A Demethylation Mediated by FTO in the Cell Nucleus and Cytoplasm. Mol Cell *71*, 973-985.e975.

Wu, Z., Shi, Y., Lu, M., Song, M., Yu, Z., Wang, J., Wang, S., Ren, J., Yang, Y.G., Liu, G.H.*, et al.* (2020). METTL3 counteracts premature aging via m6A-dependent stabilization of MIS12 mRNA. Nucleic Acids Res *48*, 11083-11096.

Wu, Z., Zhang, W., Song, M., Wang, W., Wei, G., Li, W., Lei, J., Huang, Y., Sang, Y., Chan, P.*, et al.* (2018). Differential stem cell aging kinetics in Hutchinson-Gilford progeria syndrome and Werner syndrome. Protein Cell *9*, 333-350.

Xiang, Y., Laurent, B., Hsu, C.H., Nachtergaele, S., Lu, Z., Sheng, W., Xu, C., Chen, H., Ouyang, J., Wang, S.*, et al.* (2017). RNA m(6)A methylation regulates the ultraviolet-induced DNA damage response. Nature *543*, 573-576.

Zhang, X., Liu, Z., Liu, X., Wang, S., Zhang, Y., He, X., Sun, S., Ma, S., Shyh-Chang, N., Liu, F.*, et al.* (2019a). Telomere-dependent and telomere-independent roles of RAP1 in regulating human stem cell homeostasis. Protein Cell *10*, 649-667.

Zhang, X., Wei, L.H., Wang, Y., Xiao, Y., Liu, J., Zhang, W., Yan, N., Amu, G., Tang, X., Zhang, L.*, et al.* (2019b). Structural insights into FTO's catalytic mechanism for the demethylation of multiple RNA substrates. Proc Natl Acad Sci U S A *116*, 2919-2924.

Zhang, Y., Liu, T., Meyer, C.A., Eeckhoute, J., Johnson, D.S., Bernstein, B.E., Nusbaum, C., Myers, R.M., Brown, M., Li, W.*, et al.* (2008). Model-based analysis of ChIP-Seq (MACS). Genome Biol *9*, R137.

Zhou, Y., Zhou, B., Pache, L., Chang, M., Khodabakhshi, A.H., Tanaseichuk, O., Benner, C., and Chanda, S.K. (2019). Metascape provides a biologist-oriented resource for the analysis of systems-level datasets. Nat Commun *10*, 1523.

Supplemental Figure Legends

Figure S1. Generation and characterization of *FTO*$^{-/-}$ hESCs and hMPCs.

A. Full-length western blot image showing the knockout of FTO in *FTO*$^{-/-}$ hESCs. GAPDH was used as the loading control.

B. Table summarizing that no off-target effects were identified at the top 7 predicted off-target sites by FTO-targeting sgRNA.

C. Karyotype analysis of *FTO*$^{-/-}$ hESCs.

D. Copy number variation (CNV) analysis via whole genome sequencing in *FTO*$^{+/+}$ and *FTO*$^{-/-}$ hESCs.

E. CNV analysis of the *FTO*-located chromosome (Chr. 16) in *FTO*$^{+/+}$ and *FTO*$^{-/-}$ hESCs.

F. Violin plot showing the CNV analysis at the surrounding genomic regions of the sgRNA-targeting site (~5 Kb upstream and downstream) in *FTO*$^{+/+}$ and *FTO*$^{-/-}$ hESCs. ns, not significant.

G. DNA methylation analysis at the *OCT4* promoter region in *FTO*$^{+/+}$ and *FTO*$^{-/-}$ hESCs. Six clones (n = 6) of each hESC line were picked out and processed for DNA methylation analysis.

H. RT-qPCR analysis of *OCT4*, *SOX2*, *NANOG* in *FTO*$^{+/+}$ and *FTO*$^{-/-}$ hESCs. Data are presented as the means ± SEM. n = 4 biological replicates. ns, not significant.

I. Immunofluorescence analysis of HP1α in *FTO*$^{+/+}$ and *FTO*$^{-/-}$ hESCs. Scale bars, 20 μm. Data are presented as the means ± SEM. n > 300 cells from three biological replicates. ns, not significant.

J. Immunofluorescence analysis of H3K9me3 in *FTO*$^{+/+}$ and *FTO*$^{-/-}$ hESCs. Scale bars, 20 μm. Data are presented as the means ± SEM. n > 300 cells from three biological replicates. ns, not significant.

K. Western blot analysis of LAP2 and HP1α in *FTO*$^{+/+}$ and *FTO*$^{-/-}$ hESCs. GAPDH was used as the loading control. Data are presented as the means ± SEM. n = 3 biological replicates. ns, not significant.

L. Flow cytometric analysis of hMPC-specific markers including CD73, CD90 and CD105 in *FTO*$^{+/+}$ and *FTO*$^{-/-}$ hMPCs. Scale bars in phase images, 100 μm.

M. Full-length western blot image showing the knockout of FTO in *FTO*$^{-/-}$ hMPCs. GAPDH was used as the loading control.

N. Analysis of the adipogenesis capacity of *FTO*$^{+/+}$ and *FTO*$^{-/-}$ hMPCs by Oil Red O staining. Scale bars, 50 μm. Data are presented as the means ± SEM. n = 3 biological replicates. *, $P < 0.05$.

O. Analysis of the chondrogenesis capacity of *FTO*$^{+/+}$ and *FTO*$^{-/-}$ hMPCs. Representative images for bright filed and Toluidine Blue O staining were shown. Scale bars, 50 μm. The relative diameters of chondrospheres were determined and data are presented as means ± SEM. n = 3 biological replicates. ns, not significant.

P. Analysis of the osteogenesis capacity of *FTO*$^{+/+}$ and *FTO*$^{-/-}$ hMPCs by von Kossa staining. Scale bars, 100 μm. Data are presented as the means ± SEM. n = 3 biological replicates. *, $P < 0.05$.

Figure S2. Analyses of m^6A, m^6A_m and m^1A modifications in *FTO*$^{+/+}$ and *FTO*$^{-/-}$ hESCs and hMPCs.

A. Dot blot analysis of the m^6A level in total RNA extracted from *FTO*$^{+/+}$ and *FTO*$^{-/-}$ hESCs. Methylene blue (MB) staining was used as RNA loading control. Data are presented as the means ± SEM. n = 3 biological replicates. ns, not significant.

B. Dot blot analysis of the m^6A level in mRNA extracted from *FTO*$^{+/+}$ and *FTO*$^{-/-}$ hESCs. MB staining was used as RNA loading control. Data are presented as the means ± SEM. n = 3 biological replicates. ns, not significant.

C. LC-MS/MS analysis of the m^6A level in mRNA extracted from *FTO*$^{+/+}$ and *FTO*$^{-/-}$ hESCs. Data are presented as the means ± SEM. n = 3 biological replicates. ns, not significant.

D. Dot blot analysis of the m^6A level in total RNA extracted from *FTO*$^{+/+}$ and *FTO*$^{-/-}$ hMPCs. MB staining was used as RNA loading control. Data are presented as the means ± SEM. n = 3 biological replicates. ns, not significant.

E. Dot blot analysis of the m^6A level in mRNA extracted from *FTO*$^{+/+}$ and *FTO*$^{-/-}$ hMPCs. MB staining was used as RNA loading control. Data are presented as the means ± SEM. n = 3 biological replicates. ns, not significant.

F. LC-MS/MS analysis of the m^6A level in mRNA extracted from $FTO^{+/+}$ and $FTO^{-/-}$ hMPCs. Data are presented as the means ± SEM. n = 4 biological replicates. ns, not significant.

G. Fluorometric assay for detection of the m^6A abundance in total RNA (left) and mRNA (right) extracted from $FTO^{+/+}$ and $FTO^{-/-}$ hESCs. Data are presented as the means ± SEM. n = 4 biological replicates (left) and n = 3 biological replicates (right). ns, not significant.

H. Fluorometric assay for detection of the m^6A abundance in total RNA (left) and mRNA (right) extracted from $FTO^{+/+}$ and $FTO^{-/-}$ hMPCs. Data are presented as the means ± SEM. n = 3 biological replicates. ns, not significant.

I. Heatmap showing the correlation of RNA-seq (input) and MeRIP-seq replicates in $FTO^{+/+}$ and $FTO^{-/-}$ hESCs. The color keys from blue to red represent the similarity (Pearson’s correlation) from low to high.

J. Heatmap showing the correlation of RNA-seq (input) and MeRIP-seq replicates in $FTO^{+/+}$ and $FTO^{-/-}$ hMPCs. The color keys from blue to red represent the similarity (Pearson’s correlation) from low to high.

K. Cumulative curves showing normalized $\log_2$ peak intensity of m^6A in transcripts from $FTO^{+/+}$ and $FTO^{-/-}$ hESCs.

L. Cumulative curves showing normalized $\log_2$ peak intensity of m^6A in transcripts from $FTO^{+/+}$ and $FTO^{-/-}$ hMPCs.

M. LC-MS/MS analysis of the m^6A_m level in mRNA extracted from $FTO^{+/+}$ and $FTO^{-/-}$ hESCs. Data are presented as the means ± SEM. n = 3 biological replicates. ns, not significant.

N. LC-MS/MS analysis of the m^6A_m level in mRNA extracted from $FTO^{+/+}$ and $FTO^{-/-}$ hMPCs. Data are presented as the means ± SEM. n = 4 biological replicates. ns, not significant.

O. Dot blot analysis of the m^1A level in tRNA extracted from $FTO^{+/+}$ and $FTO^{-/-}$ hESCs. MB staining was used as RNA loading control. Data are presented as the means ± SEM. n = 3 biological replicates. ns, not significant.

P. Dot blot analysis of the m^1A level in tRNA extracted from $FTO^{+/+}$ and $FTO^{-/-}$ hMPCs. MB staining was used as RNA loading control. Data are presented as the means ± SEM. n = 3 biological replicates. ns, not significant.

Q. Network showing the potential FTO-interacting proteins associated with “mitotic cell cycle process” that identified by mass spectrometry analysis. The

color keys from light to dark represent low to high coverages of FTO-interacting proteins, respectively. Gene symbols of these FTO-interacting proteins are shown.

R. Table showing that MIS12 was identified as a novel FTO-interacting protein and NSL1 was identified as a candidate protein interacting with FTO by mass spectrometry analysis.

S. Western blot analysis of MIS12 in $FTO^{+/+}$ and $FTO^{-/-}$ hESCs. GAPDH was used as the loading control. Data are presented as the means ± SEM. n = 3 biological replicates. ns, not significant.

T. Integrative Genomics Viewer (IGV) plots showing the mRNA abundance of *MIS12* in $FTO^{+/+}$ and $FTO^{-/-}$ hMPCs.

U. IGV plots showing the IP (m^6A) and Input reads of *MIS12* mRNA in $FTO^{+/+}$ and $FTO^{-/-}$ hMPCs.

V. qPCR analysis of *MIS12* mRNA levels in $FTO^{+/+}$ and $FTO^{-/-}$ hMPCs. Data are presented as the means ± SEM. n = 3 biological replicates. ns, not significant.

W. MeRIP-qPCR analysis for detection the m^6A enrichment on *MIS12* mRNA in $FTO^{+/+}$ and $FTO^{-/-}$ hMPCs. Data are presented as the means ± SEM. n = 3 biological replicates. ns, not significant.

X. Western blot analysis of MIS12 in $FTO^{-/-}$ hMPCs treated with Vehicle, MG132 or BFA1. GAPDH was used as the loading control. Data are presented as the means ± SEM. n = 3 biological replicates. ns, not significant; *, $P < 0.05$.

Y. Western blot analysis of MIS12 in $FTO^{+/+}$ and $FTO^{-/-}$ hMPCs upon treatment with vehicle or MG132. GAPDH was used as the loading control. Data are presented as the means ± SEM. n = 4 biological replicates. ***, $P < 0.001$.

Z. Statistical results showing the relative expression levels of MIS12 in control (sgNTC) and MIS12-knockout (sgMIS12) hMPCs (corresponding to Fig. 2K). Data are presented as the means ± SEM. n = 3 biological replicates. **, $P <$ 0.01.

Supplemental Table Legends

Table S1. List of potential FTO-interacting proteins identified by co-IP assay followed by LC-MS/MS analysis.

Table S2. Sequence information of sgRNAs and primers used for gene editing, *OCT4* promoter amplification, plasmid construction and qPCR analysis.

Figure S1

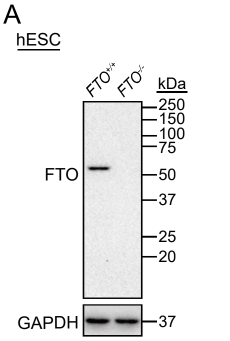

B

Potential off-target (OT) sites	Sequences (5'-3')	Locus	Off-target YES or NO
On-target	GAAGCGCACCCCGACTGCCGAGG	Chr. 16, 53704187 to 53704209	——
OT-1	CAAGCGCACCCAGACTGCCTAGG	Chr. 7, 134433029 to 134433051	NO
OT-2	CCAGGGAAGTCGGGGTGCGCTCC	Chr. 17, 57256223 to 57256245	NO
OT-3	CCCCAGCAGTCGGGGGGCGTTTG	Chr. 12, 57038479 to 57038501	NO
OT-4	GAAGCTCAGCCCGACTGCCAGGG	Chr. 8, 140429030 to 140429052	NO
OT-5	GAGGCGCCCTCCGACTGCTGGAG	Chr. 12, 102419649 to 102419671	NO
OT-6	CCTCGGCAGTCAGGGTGAGCGTG	Chr. 7, 739717763 to 73971785	NO
OT-7	CTAAGGCAGTCGGGGTGAGTGTC	Chr. 9, 36004569 to 36004591	NO

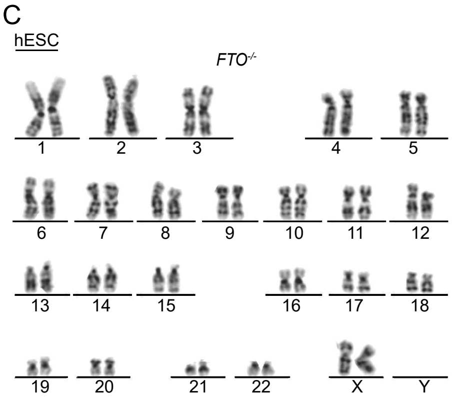

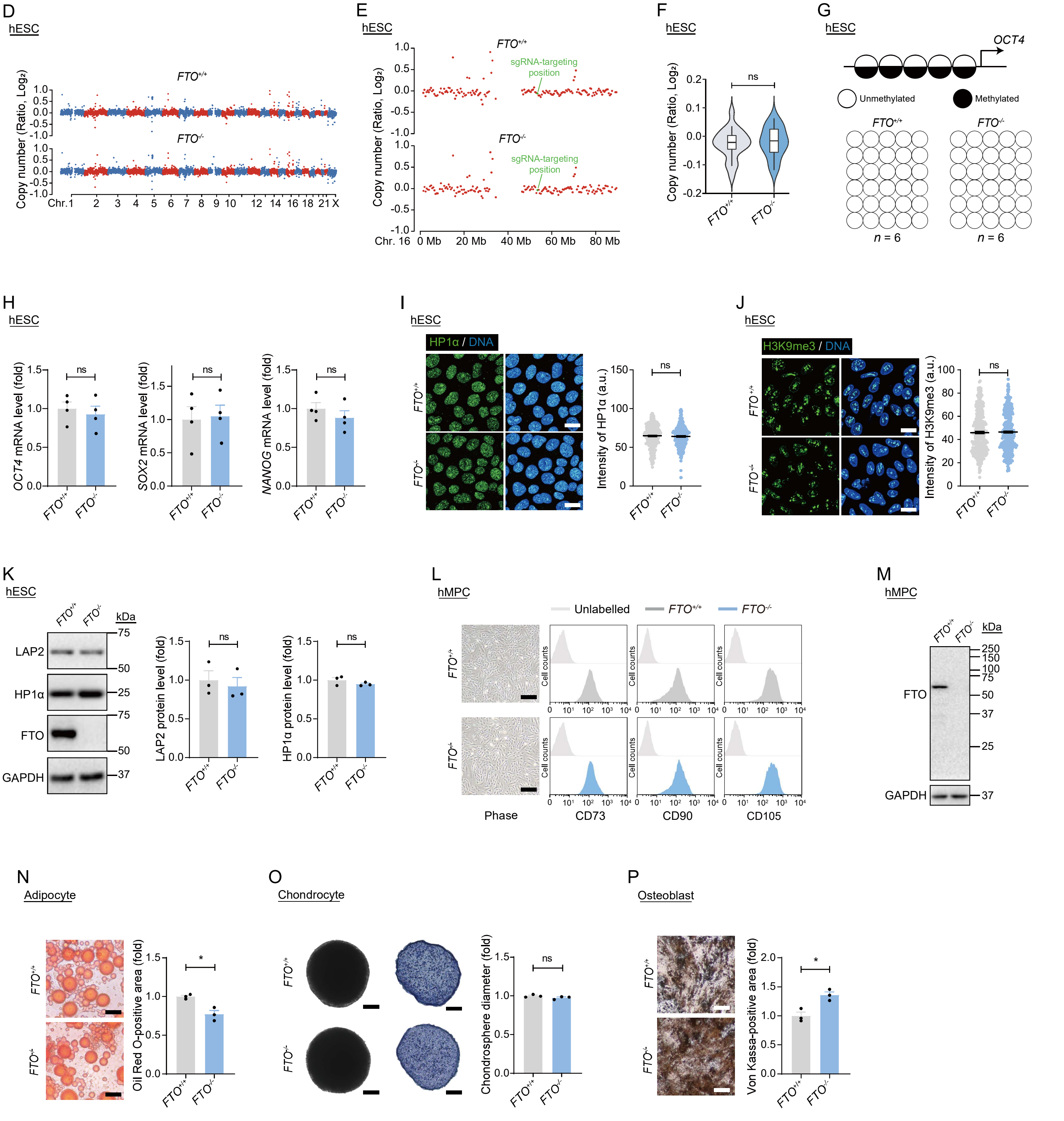

Figure S2

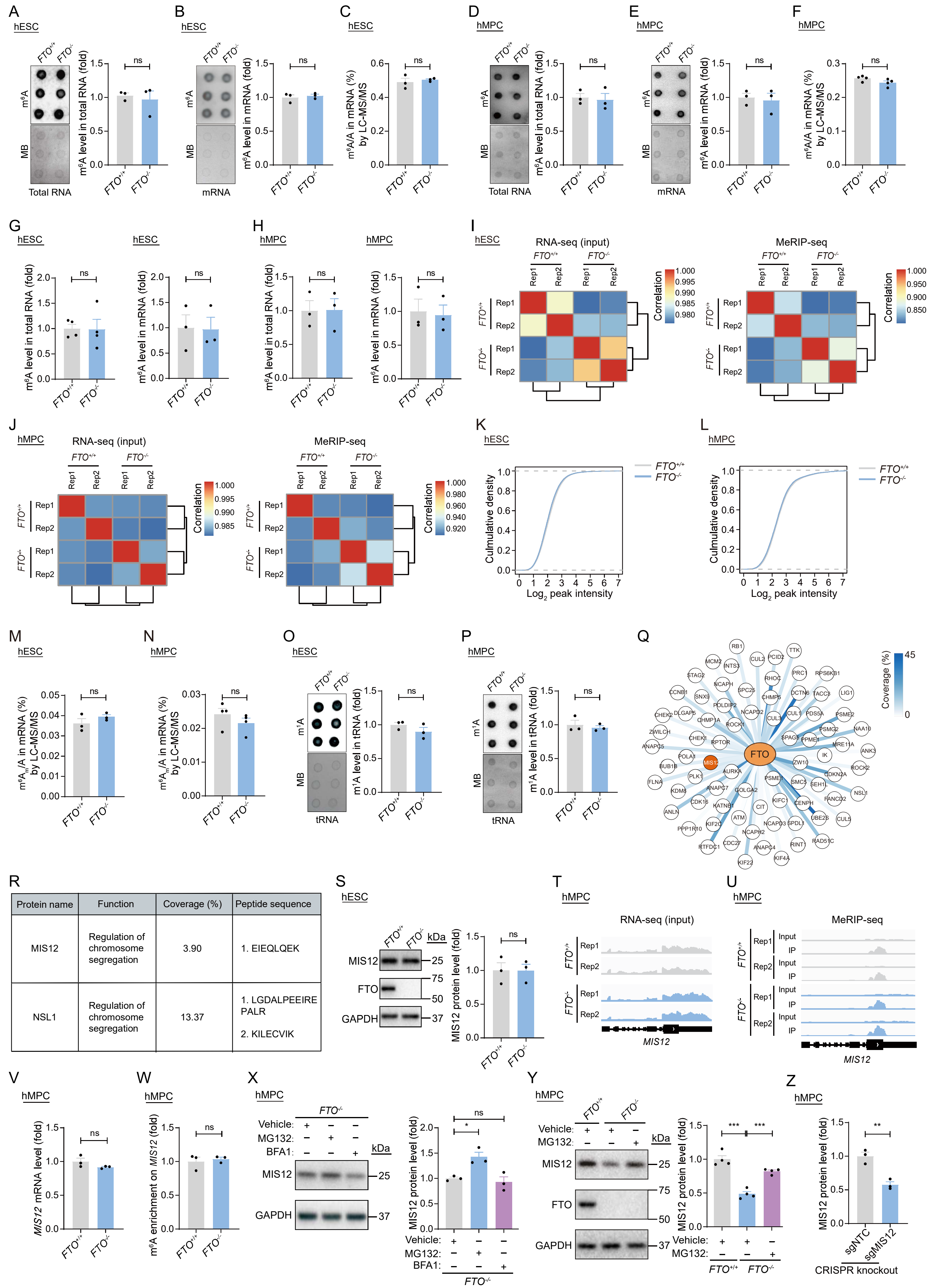

A
hESC
FTO+/+
FTO-/-
m6A
MB
Total RNA
m6A level in total RNA (fold)
ns
B
hESC
mRNA
m6A level in mRNA (fold)
C
hESC
m6A/A in mRNA (%) by LC-MS/MS
D
hMPC
E
hMPC
F
hMPC
G
hESC
H
hMPC
I
hESC
RNA-seq (input)
MeRIP-seq
Rep1
Rep2
Correlation
J
hMPC
K
hESC
L
hMPC
Culmulative density
Log2 peak intensity
M
hESC
m6Am/A in mRNA (%) by LC-MS/MS
N
hMPC
O
hESC
m1A
tRNA
m1A level in tRNA (fold)
P
hMPC
Q
FTO
MIS12
Coverage (%)
R
Protein name
Function
Coverage (%)
Peptide sequence
MIS12
Regulation of chromosome segregation
3.90
1. EIEQLQEK
NSL1
Regulation of chromosome segregation
13.37
1. LGDALPEEIRE PALR
2. KILECVIK
S
hESC
MIS12
FTO
GAPDH
kDa
MIS12 protein level (fold)
T
hMPC
RNA-seq (input)
MIS12
U
hMPC
MeRIP-seq
Input
IP
V
hMPC
MIS12 mRNA level (fold)
W
hMPC
m6A enrichment on MIS12 (fold)
X
hMPC
Vehicle:
MG132:
BFA1:
Y
hMPC
***
Z
hMPC
sgNTC
sgMIS12
CRISPR knockout
**
